# Impact and Adaptation of Reproductive Training During COVID-19 Pandemic in Malaysia

**DOI:** 10.21315/mjms2021.28.5.3

**Published:** 2021-10-26

**Authors:** Abdul Kadir Abdul Karim, Muhammad Azrai Abu, Mohd Faizal Ahmad, Norazilah Mat Jin, Haris Njoo Suharjono

**Affiliations:** 1 Department of Obstetrics and Gynaecology, Faculty of Medicine, Universiti Kebangsaan Malaysia, Cheras, Kuala Lumpur, Malaysia; 2 Department of Obstetrics and Gynaecology, Faculty of Medicine, Universiti Teknologi MARA, Sungai Buloh, Selangor, Malaysia; 3 Department of Obstetrics and Gynaecology, Sarawak General Hospital, Kuching Sarawak, Malaysia

**Keywords:** COVID-19, pandemic, reproductive medicine, training, women’s health

## Abstract

The novel coronavirus (COVID-19) pandemic has affected the community at large. It has affected almost everyone and every aspect of social, economic and educational activities. Training in reproductive medicine has not been spared, as training in this field requires a combination of clinical interaction with patients, procedural experience, constant discussions and the element of research. The changes to numbers of new infections or active cases dictate the restrictions placed on the community and health care services alike. At the beginning of the pandemic, both the patients’ fear of going to a health care facility and movement restrictions had caused a significant reduction in the number of COVID-19 cases. Furthermore, the Ministry of Health (MOH) Malaysia’s recommendation to withhold all non-essential medical services, including those related to reproductive medicine, falls under this category. Therefore, it could negatively impact the quality of training and lead to an extension of training duration in reproductive medicine. Thus, the procedural experience could be supplemented with simulator training, teleconsultation could replace standard clinic sessions and online meeting platforms could replace routine academic meetings. Any modifications must be adaptable or flexible, as similar infectious pandemics and restrictions could recur from time to time.

## Introduction

The novel coronavirus disease (COVID-19) has become a cause of concern to most societies since the first reported case in Wuhan, China (1). The description of this disease’s clinical features in a cluster of 41 patients by January 2020 was described by Huang et al. (2). COVID-19 had caused severe respiratory illness associated with high intensive care unit (ICU) admission and increased mortality. This fear has gradually spread throughout the world over a few months. In Malaysia, the disease was first detected on our shores on 24 February 2020, through an imported case (3). The spread of COVID-19 and the morbidity and mortality associated with it led to the World Health Organization (WHO) (1) declaring it a pandemic on 11 March 2020 (4). The Malaysian government took heed of this and started implementing the Movement Control Order (MCO) from 18 March 2020 (5).

The implementation of MCO had caught the public by surprise. Health care authorities had scrambled to establish specific measures to curtail the spread of COVID-19. Elective appointments and surgeries were postponed as part of a national effort. The reproductive medicine services were amongst the first to be struck with these measures. Being generally considered a non-essential service, appointments for the reproductive clinic were among the first to be curtailed. Essential treatment is a service that, if it is withheld, can be life-threatening or leads to an increase in morbidity and thus should not be delayed. Nonetheless, in reproductive medicine, there are always the unseen effects of reduced fecundity by delaying fertility treatment, especially in those at the end of the extremes of reproductive age (6) and those with a disease that impacts their ovarian reserve (7–8). Delaying fertility treatment could lead to a poorer outcome. Indirect psychological and monetary impact on delaying fertility treatment has yet to be evaluated in the literature and should not be dismissed.

However, the recognition that infertility care as an essential service was subsequently highlighted by the American Society for Reproductive Medicine (ASRM) in their second updated statement on the pandemic on 13 April 2020 (9) and also by the joint body of ASRM, European Society for Human Reproduction (ESHRE) and International Federation of Fertility Societies (IFFS) on 29 May 2020 (10). International bodies such as ESHRE (11) and ASRM (12) quickly published their guidelines and recommendations during this pandemic. The recommendations include continuing to care for patients who are currently ‘in-cycle’ or who require urgent stimulation and cryopreservation; to suspend initiation of new treatment cycles, including ovulation induction, intrauterine inseminations (IUIs), in vitro fertilisation (IVF)/ intra-cytoplasmic sperm injection (ICSI) such as retrievals and frozen embryo transfers and non-urgent gamete cryopreservation; to consider cancellation of all embryo transfers whether fresh or frozen; to suspend elective surgeries and non-urgent diagnostic procedures, and to minimise in-person interactions and increase utilisation of telehealth.

In essence, the advice was to postpone all artificial reproductive technology (ART) (13) cycles that had not started and to avoid doing fresh transfers for IVF/ICSI cycles. Although there was evidence that full-term babies delivered from mothers with active disease have performed well (13), severe forms of COVID-19 infection may precipitate preterm labour (14–15). Reassuringly, infected mothers with ceasarean section delivery also did not show the presence of viral RNA in the amniotic fluid, cord blood or breast milk (16). Although the evidence of vertical transmission is still unclear, the main concerns were if it was to be proven, its impact on the foetus during the first or second trimester of pregnancy might not be evident for the next few months.

Malaysia has undergone three phases of MCO, including four phases of MCO from 18 March until 4 May, followed by the Conditional MCO (CMCO) that lasted until 9 June and the Recovery MCO (RMCO), which is due to end on the 31 December 2020. Several states had since been placed back under the CMCO due to the increase in cases. Travel, social gatherings and business activities were significantly restricted during the MCO, and this was gradually relaxed during the subsequent CMCO and RMCO. Thus far, Malaysia has done tremendously well in mitigating and managing this pandemic compared to other nations such as the United States, India and a few Latin American countries. Despite the uncertainties in the COVID-19 situation in Europe and the United States, ESHRE in their phase two update on assisted reproduction and COVID-19, on the 23 April (17), and subsequently ASRM in their third update on recommendations during the COVID-19 pandemic on the 24 April, provided guidelines on resuming care (18).

The objective of this review is to elaborate on the impact of the COVID-19 pandemic on the reproductive training at our centre and the adaptation implemented for the enforced changes by the current situation. There is currently paucity in the literature regarding the impact of the COVID-19 pandemic on reproductive training during this period. Hence, we described the impact that the pandemic has had on reproductive training at our centre by explicitly relating it to the trainers, trainees, consultations, procedures, research, journal clubs and proceedings, and congresses.

## International Guidelines on Reproduction Services During Pandemic

This pandemic is unprecedented in this modern age of health care of numerous guidelines from international governing bodies. The situation is ever so volatile and dynamic that multiple statements and guidelines from various clinical bodies are being produced at an unprecedented rate, clearly demonstrated by the plethora of guidelines updates. At present, there are five updates for the recommendations by ASRM (9, 12, 18–21), while ESHRE has produced two further statements regarding assisted reproduction and COVID-19 after their initial statement (11, 17, 22).

## Reproductive Training Centre

The Advanced Reproductive Centre in Hospital Canselor Tuanku Muhriz has long been a recognised training centre for reproductive training for clinicians and allied health personnel. The centre provides training for trainees from the Ministry of Health (MOH) Malaysia, various universities under the Ministry of Higher Education (MOHE), international clinicians, private institutions employees and self-funded candidates. Our centre performs an average of 300–400 fresh IVF/ICSI cycles annually and 200–300 IUI cycles per year over the last 5 years. The centre also recently provides oncofertility services, which, despite requiring urgency due to gonadotoxic chemotherapy required, was also reduced during this pandemic (23). A total of almost 10,000 patients visit our reproductive centre, which includes new case appointments, follow-ups, follicular tracking and procedural cases. The centre is well staffed and equipped to provide the current load of cases with sufficient time allocated for teaching and supervisor-fellow interaction. We have three consultants who are reproductive experts practicing at our centre with four full-time embryologists.

## General Measures for Pandemic

### Patients

All appointments at the clinic and those on the ongoing fertility treatment cycle were contacted and given an option to postpone their appointment or cycles in line with the international recommendations (11–12). Those who opted to continue with treatment could do so after consultation. Patients entering our clinic needed to pass a questionnaire screening for risk factors and thermal checks. Moreover, usage of a facemask was made mandatory upon entry.

### Healthcare Personnel

All staff and trainees were also subjected to the same requirement of thermal screening on a daily basis. Face shield or goggles and a disposable gown also needed to be worn during interaction with patients in the clinic as per hospital requirements.

### Trainers

All the consultants and embryologists were made aware of restrictions to reproductive services in view of the recommendations of international bodies and the MOH. There were also restrictions on movements that were being enforced to consider. The travel bans during MCO did not impair them from being present to provide direct guidance to trainees. The only adaptation they had to make as trainers is the limitation in the number of trainees for in-clinic consultation and procedural cases. The number of trainees allowed to be in attendance during consultation and procedure was reduced to only one from the usual two to comply with social distancing measures.

### Trainees

#### Movement

At the onset of the MCO, the centre had three clinical fellows, two from the MOHE and one from MOH, and one allied health personnel from a private sector attached to the centre for training. An international clinical fellow was scheduled to arrive in April 2020; however, due to the closure of our country’s borders, the candidate’s attachment had to be postponed. A two-week quarantine order was also made mandatory for one of the trainees due to recent travel to a high-risk country. The international travel restriction has had a minor impact on the trainees from overseas. This effect would be similar to all international students and foreign workers. Possible alternative measures are online registration for foreign candidates; however, this is far from ideal for reproductive training as performing procedures still have hands-on requirements. For local trainees, this is not expected to have an effect.

#### Placement

The reproductive centre receives numerous requests for attachment for inspiring reproductive specialist. As such, the centre needs to juggle the schedules of clinical fellows carefully to maximise training that can be provided. We have been collaborating with MOH for the training of reproductive medicine specialists. There is a constant presence of trainees from the MOH as part of 3 years training programme required for the reproductive medicine sub-specialty. At any single time, there would be one or two candidates from MOH spending 6 months–1 year at this centre. As part of a national-level preparedness for the worsening of the pandemic, there was a suspension in MOH training duration and a recall to their parent hospital that disrupted the planning of numbers of trainees present. The centre can only cater to a maximum of four persons at any given time point based on our number of reproductive cycles, consultant trainers and working space. As the centre also receives candidates from MOHE and overseas, careful adjustments need to be made to maintain the equilibrium. In preparedness for the future redeployment of MOH trainees, training that can be done off-site needs to be considered to maintain the scheduling of trainees currently attached to the Advanced Reproductive Centre.

## Consultation

### Clinic

Due to the travel restriction and fear amongst patients, the attendance in the clinic had seen almost 80%–90% drop in the initial phase of the MCO. The reduction had severely impacted training exposure in terms of patient counselling and case management. However, the number of patients attending the clinic gradually improved during the CMCO and reached normal levels once the RMCO was implemented on 10 June 2020 (24). Plans were made to commence teleconsultation once the Malaysian Medical Counsel (MMC) published their guidelines on virtual consultation (25), which would have helped the trainees’ exposure. Although it was not implemented as the attendance started to improve, this plan is in place if the country reimplements the travel restrictions. During a consultation, the number of trainees is also limited in the consultation room to enable social distancing. In addition, personal protective equipment of gown, facemask and a face shield needs to be worn in line with the hospital infection prevention and control guidelines.

## Ongoing Cycle

In line with international bodies’ recommendations (11–12), patients with ongoing cycles at the beginning of the MCO were advised to abandon the cycle and freeze all embryo for IVF/ICSI cycles. Patients that continued their treatment cycle and those that subsequently began their ART cycle were fully aware of the unknown future risk to the pregnancy should they become infected after a successful cycle. The reduction in ongoing cycles meant reduced exposure in managing the different responses in a stimulated ART cycle and the procedure’s hands-on training. Performing IUI, oocyte retrieval and embryo transfer is a skill that is essential for all reproductive trainees. In the event of prolonging MCO enforcement and reducing procedures, other options of training had been considered. One such option is to purchase simulation models such as PickUpSim^TM^ oocyte retrieval simulator (26), VirtaMed GynoS^TM^ ASRM embryo transfer simulator (27) is available in the market. Soave et al. (28) had performed a study that included 44 clinicians and showed that simulator-based training for oocyte retrieval was effective for novice clinicians (28). The lack of IUI procedures would not pose a similar problem, as the insertion of an IUI catheter is similar to inserting a Pipelle de Cornier® endometrial sampler, which is a standard procedure for gynaecologist. However, there is no substitute for performing the actual procedure and simulator base training should be reserved for a situation when there is a lack of patients.

## Procedural Training

Opportunity to perform oocyte retrieval, embryo transfer and IUI is all affected due to the reduction in numbers. The oocyte retrieval procedures are done using sedatives, while embryo transfer and IUI does not require analgesia. No aerosol risks are involved in the procedures. As such, the main concern was the exposure to the bodily fluids of semen and follicular fluids. There has been evidence regarding the presence of COVID-19 antigen in seminal fluid (29). However, the capacity for it to infect a health care personal is yet to be proven. There has been no evidence that COVID-19 antigen is present in the follicular fluid at present. A study is scheduled to be performed on this possibility, and the reproductive community is eagerly awaiting the results (30). Standard use of aseptic technique and sterile equipment is sufficient during the procedure. If there was an IUI, oocyte retrieval, IVF, ICSI and embryo transfer, no change to our current practice was deemed necessary and standard aseptic technique using similar protective equipment prior to the pandemic was sufficient. The approach is similar to the recommendation by ASRM (19).

## Journal Club

Journal club is an essential part of training to keep abreast of the current developments and evidence-based practices. Trainees need to be taught to critically appraise the multitude of evidence in this current age and its relevance to their practice. This educational format was uninterrupted during this pandemic. However, for the future restriction that may prevent the trainees from being on-site, online platforms such as Zoom, Microsoft Teams and Google Meet provide a suitable alternative for the longevity of this method of training.

## Research and Publication

Conducting and understanding the value of research is an essential part of reproductive training. It provides valuable training for trainees to appreciate the depth and technicality of articles produced to guide us in our reproductive practices. Trainees that do prospective clinical research on patients will have to extend the duration of trials or even consider changing the type of research done due to a reduction in the number of patients within the time frame of the research period.

## Proceedings and Congresses

Presentation at proceedings and attendance at congress is encouraged for reproductive trainees as part of their training. Unfortunately, the norm of large gatherings for on-site proceedings and congress grinds to a halt during this pandemic. However, we are blessed with the current advancement in technology with seamless conversion to webinars and online congress.

## Future

In light of the lessons learnt and the uncertainties of the COVID-19 pandemic in Malaysia, the MOH’s mantra, *‘*prepare for the worst and hope for the best’, needs to be put into practice for potential future pandemics. A systematic and flexible plan that can be implemented based on such scenarios needs to be appropriately discussed, planned and it has to be practical. Amongst our preliminary plans that have been discussed yet implemented due to the improvement in a situation were teleconsultation, using simulators for procedures, online registrations for training and creating learning outcome goals for off-site training. This may yet become a reality as suggested in the ASRM latest update on 23 June 2020, where the body has acknowledged that the reproductive medicine community will need to continue their practice in a COVID-19 environment at least until an effective vaccine or effective treatment becomes widely available (21).

## Conclusion

The current evidence of viral transmission of COVID-19 has had an impact on training in reproductive medicine. Various restrictions to minimise contact with our clients have forced us to re-evaluate our traditional strategies of training. If we fail to adapt to the new social norm, there is a possibility that we will reduce the quality of training that can have an impact on future reproductive services. The improvement made needs to be guided by evidence-based data and expert opinions on the effect of adaptations made for reproductive training, which is currently lacking.
